# Creating partnerships between veterinarians and underserved producers: getting started with establishing veterinary client–patient relationships

**DOI:** 10.3389/fvets.2025.1595301

**Published:** 2025-07-09

**Authors:** Nicola L. Ritter, Torri Whitaker, Molly Gonzales, Glennon Mays, Kevin Eugene Washburn, Richard D. Posey

**Affiliations:** ^1^College of Veterinary Medicine & Biomedical Sciences, Texas A&M University, College Station, TX, United States; ^2^Texas A&M AgriLife Research, College Station, TX, United States; ^3^Department of Large Animal Clinical Sciences, West Texas A&M University, Canyon, TX, United States

**Keywords:** veterinarian-producer partnership, veterinary-client-patient-relationship, education programs, operation sustainability, communication, online training

## Abstract

**Objectives:**

To provide local veterinarians and producers opportunities for collaboration and applied education related to the Veterinary Client Patient Relationship (VCPR); to assess the effectiveness of such opportunities in enhancing VCPR knowledge and confidence; and to establish VCPRs in hopes of improving economic efficiency and sustainability *of small to medium-sized livestock operations.*

**Materials and methods:**

Two one-day “Creating Partnerships” workshops utilized educational resources in five learning sessions focused on communication, establishing a Veterinarian Client-Patient Relationship (VCPR), and telemedicine. A pre- and post-test, comprised of closed-ended and Likert scale questions, measured the workshops’ effectiveness in increasing knowledge and confidence related to veterinary and producer partnerships.

**Results:**

Pre- and post-test scores indicate significant increases in knowledge and confidence related to Veterinary Client-Patient Relationships. Twenty-seven percent of participants established a Veterinary Client-Owner-Patient Relationship (VCPR) with their local veterinarian following the workshops.

**Discussion:**

Targeted educational outreach shows promise in creating and maintaining veterinarian-producer relationships. Knowledge gains and attitude shifts related to Veterinary Patient Client Relationships may improve animal health outcomes, resulting in more sustainable and profitable livestock operations and veterinary practices.

## 1 Introduction

Small and medium-sized cattle operations represent a significant portion of the U.S. livestock industry, particularly in rural and underserved regions. These operations are often family-owned and managed with limited resources, making them vital to local economies and food systems. Despite their importance, they frequently face systemic barriers to accessing consistent veterinary care, which can compromise animal health, biosecurity, and overall farm sustainability.

The Veterinary-Client-Patient Relationship (VCPR) is integral to animal health and well-being, and can improve the sustainability and profitability of both veterinary practices and livestock operations. Established when a veterinarian agrees with a client to provide veterinary services to an animal or group of animals of which he has sufficient knowledge, and often accompanied by a written agreement, the VCPR identifies the veterinarian as responsible for treatment protocols, prescriptions, personnel training, oversight, and drug use in livestock operation (1). Ritter et al. (2) found that both livestock producers and veterinarians noted trust, communication, and education as key components to creating and sustaining such a relationship. Although discussions about the benefits of VCPRs occur in both producer and veterinary professional organizations, actionable steps leading to quantifiable outcomes have yet to emerge (3). Building on descriptions of barriers and the willingness to establish veterinarian and producer partnerships gained from our previous study, we aim to develop feasible partnership pathways through educational resources and experiential opportunities.

Establishing a VCPR is particularly challenging for small and medium-sized producers due to a combination of systemic and logistical barriers. Many rural areas face a shortage of large animal veterinarians, limiting producers’ ability to initiate or maintain a VCPR. Economic constraints further complicate this issue, as producers often weigh the cost of veterinary services against the market value of individual animals, resulting in delayed or forgone care unless absolutely necessary. Additionally, a mutual perception gap exists: veterinarians may feel undervalued by producers, while producers may perceive veterinarians as inaccessible or dismissive of their operational needs. Time and logistical challenges also play a significant role, as the on-site visits required to establish a valid VCPR in most states are often difficult to coordinate and sustain for both parties.

### 1.1 Trust as a component of time

Both veterinarians and producers indicate time as a significant barrier to relationship development and maintenance, whether it is the time required for a veterinarian to make a farm call or for producers to connect with a veterinarian (2). According to the Texas State Board of Veterinary Medical Examiners, a veterinarian-client-patient relationship may not be established solely through telemedicine. Thus, allocating time for regular site visits is an essential component of a valid VCPR. Further, studies by Grant (4) and Sumner et al. (12), indicate producers’ trust is gained when veterinarians get to know their clients and operations. This trust is often translated into producers implementing veterinarian recommendations. As veterinarians continue to invest time in understanding their clients’ operations, their recommendations prove more effective, and the producer’s trust in them grows.

### 1.2 Communication based on trust

Trust, engendered by taking time to ‘get to know one another,’ promotes ‘cooperative discussions’—reciprocal conversations where producers offer information about their operation. This leads to shared decision-making and joint construction of feasible plans (4). Herd health programs are only successful when they meet the specific needs of individual producers; therefore, veterinarians and producers need to engage in goal-oriented dialogue (5). Such well-structured, efficient conversations tend to result in plans tailored to an individual producer’s operation, which improves the effectiveness of implemented veterinary recommendations (4). This intentional communication then transforms the overly common emergency, `fire-engine medicine’ approach to opportunities for consulting, advising, guiding, and supporting clients regarding the health and welfare of their herd (6).

Ongoing communication in the form of consultation and advising also improves producer morale. Dairy farmers participating in a calf benchmarking project with their veterinarians reported increased motivation to improve their calf management practices (12). This motivation to implement change, which improves animal welfare and economics, catalyzes successful herd health systems (5). Conversely, communication breakdowns—such as directive or paternalistic styles that fail to elicit producer input—can hinder collaboration and reduce the likelihood of adopting veterinary recommendations (13, 14). Effective communication involves mutual respect, shared decision-making, and an understanding of the producer’s broader goals and context (4). When veterinarians engage in transparent, honest, two-way dialogue, they are more likely to build enduring partnerships that support proactive animal health management (2).

### 1.3 Education flows from communication

Producers regard veterinarians as a trusted source of information (7). In fact, Sumner et al. (12) found that producers view veterinarian expertise as more reliable than information from other people or print and online sources. These findings agree with the needs assessment survey conducted by Ritter et al. (2) in which 48% of producers identified their veterinarian as their primary information source for animal health. Given their substantial influence, it is logical to utilize veterinarians in an educational capacity (7).

The necessity for veterinarian and client education partnerships is demonstrated in that a high percentage of producers (>70%) indicated that they were unfamiliar with clinical signs of serious cattle diseases or that cattle were at greater risk of having a disease (9). A majority of veterinarians interviewed by Ritter et al. (2) expressed similar concern about producers’ varied levels of knowledge and skills, stating, `There needs to be a fair amount of education pushed {provided to the client by their veterinarian}.’

Simultaneously, evidence suggests that producers and veterinarians are open to learning from each other and would welcome educational opportunities. For instance, both producers and veterinarians expressed openness to learning from one another, particularly in areas of disagreement such as pain management, suggesting a mutual interest in continued education (8). Producers already view their veterinarians as educators, sources of information, training, and guidance. Specifically, producers valued veterinarians as trusted educators who provided guidance on calf management, including nutrition, disease prevention, and pain mitigation (12). Additionally, Ritter et al. (2) reported that producers advocated for semi-annual or annual meetings with veterinarians to discuss regional animal health issues, emphasizing the importance of structured educational interactions. Moreover, according to Delgado et al. (9), producers prefer to receive animal-health-related information from their veterinarians. In fact, they regard veterinarians as primary translators of information, particularly scientific information (10, 12). This information is valuable for improving their knowledge and skills (2). Providing educational opportunities that allow for the integration of science and producers’ knowledge and experience leads to the development of credible and practical herd health recommendations (11).

A veterinarian-client partnership, developed through trust, communication, and education, has great potential for improving herd health and, consequently, profitability and sustainability for livestock operations and veterinary practices. The aim of this project is to create a pathway to this partnership through the development and delivery of educational resources addressing the role of the VCPR in communication, prescribing medications, and telemedicine as well as experiential opportunities applying newly acquired knowledge and skills.

Given the challenges faced by small and medium-sized cattle producers, they represent an ideal target audience for educational interventions designed to foster Veterinarian-Client-Patient Relationships (VCPRs). The workshops described in the manuscript were specifically developed to address these gaps by providing accessible education on the benefits and requirements of VCPRs, promoting trust-building and effective communication between veterinarians and producers, and offering incentives to encourage the establishment of formal veterinary relationships. By focusing on this demographic, the project meets a critical need in veterinary public health and contributes to the long-term sustainability of both livestock operations and rural veterinary practices.

## 2 Methods

### 2.1 Study overview

A multidisciplinary team from Texas A&M AgriLife Research, with collective expertise in agriculture economics, food animal medicine, curriculum development, educational technologies, and extension research and outreach, designed educational resources to address partnership barriers between livestock producers and veterinarians as identified in a needs assessment (2). Two one-day `Creating Partnerships’ workshops hosted at Prairie View A&M University (PVAMU) and 100 Ranchers (a nonprofit organization connecting people and projects within the agricultural community, particularly supporting African American ranchers) utilized these resources in five learning sessions delivered by clinicians from Texas A&M University’s College of Veterinary Medicine and VERO (Veterinary Education Research & Outreach). Session one focused on communication, establishing a Veterinarian Client Patient Relationship (VCPR), and telemedicine.

Participants included underserved livestock producers of small and medium-sized farms, veterinarians, extension professionals, and students pursuing veterinary or animal science careers. Recruitment flyers (Appendix A) were distributed through the PVAMU website, 100 Ranchers, and professional networks of extension and veterinarians. Underserved livestock producer is defined as individuals who belong to groups that have been subjected to racial or ethnic prejudice because of their identity as members of that group, without regard to their individual qualities, including Blacks or African Americans, Hispanics, and women. Although the USDA defines small and medium-sized (midsize) farms based on Gross Cash Farm Income (GCFI), which includes income from crop and livestock sales, government payments, and other farm-related income. According to the USDA, small farms are defined as those with gross cash farm income (GCFI) of less than $350,000, while mid-sized farms have a GCFI between $350,000 and $999,999. This study found that almost all producers were hesitant to share their GCFI with the research team. Instead, producers shared herd count and acreage. For this sample, the producer’s livestock operations consisted of an average of 60 animals, with most having more than one species present, and 164 acres of land, owned or leased. Given these counts, the research team expects these producers to meet the USDA’s small and medium-sized (midsize) farms criteria.

At the conclusion of the workshop, participants were asked to establish a VCPR with their local veterinarian, as findings from the needs assessment demonstrated that establishing a VCPR was the first (missed) step in creating partnerships. Using a standardized validation form (Appendix B), producers and veterinarians documented their mutual agreement, certifying that a new VCPR had been initiated. The form collected contact information for both parties and required signatures to confirm the relationship. Producers and veterinarians signed the form after the VCPR appointment was completed and a VCPR was established. Producers and veterinarians who returned the signed VCPR validation form within 2 months of the workshop were provided a $250 to encourage timely participation and enhance participant involvement and offset costs associated with a VCPR appointment. Participants who did not return signed forms received up to three follow-up phone calls over a 6-week period. Research staff used a standardized script to determine whether participants had an existing Veterinarian-Client-Patient Relationship (VCPR). Based on their responses, follow-up questions were tailored to assess the timing of VCPR establishment (before or after the workshop), encourage submission of a VCPR validation form, and promote continued engagement. For those without a VCPR, questions explored intentions to establish one and, if not, reasons for opting out. All participants were invited to future training events and given the opportunity to opt in to a contact list. Educational resources created for the workshops have been disseminated through the project website (https://www.tamucet.org/work/creating_partnerships/) and Extension Foundation, the United States Cooperative Extension System online resource-sharing platform (https://campus.extension.org). Table 1 displays the topic and objectives of each session.

**Table 1:** Statistical significance between pre-test and post-test scores.

Question	Pre-test		Post test		Pair samples *t*-test			Effect size
	Mean	SD	Mean	SD	*t*	df	*p*	Cohen’s *d*
How confident are you in applying communication strategies to communicate clearly with your local clients and navigate conflict? (*n* = 25)	4.00	0.76	4.36	0.64	2.09	24	0.02	0.86
How confident are you in defining what a Veterinarian- Client-Patient Relationship (VCPR) is? (*n* = 26)	3.23	1.18	4.38	0.64	5.43	25	<0.001	1.08
How confident are you in comparing and contrasting the role of the producer and veterinarian in establishing a VCPR? (*n* = 26)	3.08	1.02	4.27	0.78	4.93	25	<0.001	1.23
How confident are you in listing advantages of establishing a VCPR? (*n* = 26)	3.31	1.26	4.54	0.65	4.92	25	<0.001	1.28
How confident are you in listing consequences of not having a VCPR established? (*n* = 25)	3.28	1.37	4.48	0.65	4.65	24	<0.001	1.29
How confident are you in identifying the limitations for the veterinarian to provide care if a VCPR is not established? (*n* = 26)	3.31	1.29	4.12	1.03	3.25	25	0.003	1.27
How confident are you in describing how the presence or absence of a VCPR relationship impacts access to antibiotics and other pharmaceuticals for animal health? (*n* = 25)	3.50	1.36	4.38	0.57	3.17	25	0.004	1.42
How confident are you in describing how the presence or absence of a VCPR relationship impacts access to and the use of medicated feed? (*n* = 25)	3.08	1.44	4.36	0.64	4.31	24	<0.001	1.49
How confident are you in identifying strategies that can be used to provide information to the producer when utilizing distant health services? (*n* = 26)	3.46	1.10	4.38	0.64	3.83	25	<0.001	1.23
How confident are you in listing reasons to explore distant animal care? (*n* = 26)	3.24	1.27	4.32	0.75	3.75	25	<0.001	1.44
I have the knowledge and tools necessary to establish a VCPR with a client. (n = 26)	3.54	1.07	4.42	0.50	0.45	25	<0.001	1.07
I have a thorough understanding of the Feed Directive and how it supports livestock production. (*n* = 26)	4.46	1.53	4.31	0.62	0.78	25	0.31	1.54
I have a clear understanding of when distant animal care can be used and not used. (*n* = 26)	3.81	0.63	4.35	0.63	3.38	25	0.001	0.811
I have a clear understanding of how a VCPR is required to access antibiotics and other pharmaceuticals. (*n* = 26)	3.81	0.75	4.35	0.56	3.20	25	0.002	0.859
I can define the producer and veterinarian’s role when establishing a VCPR. (*n* = 26)	3.69	1.12	4.54	0.65	4.28	25	<0.001	1.01

### 2.2 Study evaluation

Researchers employed a pre- and post-test design to measure the workshops’ effectiveness in increasing knowledge and confidence related to veterinary and producer partnerships. Both assessments consisted of closed-ended and Likert scale questions structured into five sections corresponding to the workshop sessions (Appendix C). Participants completed the pretest before the workshop commenced and the posttest immediately after the final session.

## 3 Results

The workshops were attended by 45 individuals, comprising 26 livestock producers, eight pre-veterinary students, four extension professionals, four veterinarians, and three study staff. Given that the study focused on producers and veterinarians, these attendees were asked to complete a pre-test and a post-test. Of the 26 producers present, 25 completed both pre- and post-tests, 96% response rate. The four veterinarians in attendance did not complete the pre- and post-tests. A paired sample *t*-test was conducted to determine whether there were significant differences between participants’ pre-test and post-test scores. The sample consisted of 26 participants with a mean pre-test score of *M* = 66.38 (SD = 14.44) and a mean post-test score of *M* = 82.85 (SD = 9.35). The results indicated a statistically significant increase in scores from the pre-test to the post-test, *t*(25) = −5.870, *p* < 0.001. The effect size, measured by Cohen’s *d*, was calculated to be 14.30, indicating a statistically and practically significant effect. Table 2 presents the means and standard deviations for the pre-test and post-test scores.

**Table 2:** List of sessions and objectives of creating partnerships workshop.

Session	Communication: a vital role in establishing a viable relationship	The Veterinarian-Client-Patient Relationship (VCPR) mystery	Benefits of a Veterinarian-Client-Patient Relationship (VCPR) for the use and accessibility of drugs	Utilize the veterinary feed directive and additional guidelines in establishing a VCPR	Distance animal care
Objectives	Rules of engagement Rapport Your five needs Five new communication Methods Conflict resolution	Definition Establishing a VCPR Roles of veterinarian & producer Benefits Limitations	Drug residue Antibiotic Resistance Drug accessibility	Purpose Veterinarian & producer roles Uses	VCPR Reasons Type of care Techniques

Analysis of Likert questions shows an equally significant increase in confidence or strong agreement from pre to post-test across all measured areas, with highly significant *p*-values (*p* < 0.001) in many cases. Participants reported higher confidence in defining the Veterinary Client Patient Relationship (VCPR) and understanding the roles of veterinarians and producers in establishing VCPR, with large effect sizes (Cohen’s *d* ranging from 0.86 to 1.49) (Table 2). Overall, the data suggest that the workshops were highly effective in enhancing participants’ confidence and knowledge, with large effect sizes indicating meaningful changes despite the small sample size.

Of the 26 producers who attended the workshops, 27% (*n* = 7) established a VCPR with their local veterinarian. Follow-up communication was initiated for 16 participants who indicated an intention to create a VCPR with their local veterinarian after the workshop but did not return a form. Of these individuals, seven had either given incorrect contact information or did not answer the calls, six stated their intention to create a VCPR and return the form by the deadline—but never did, one had been unable to find a veterinarian whose practice included cattle and sheep, one had a VCPR in place before the workshop, and one stated that he was ineligible for our study.

## 4 Discussion

The `Creating Partnerships’ workshops had a significant positive impact on participants’ learning, as suggested by both quantitative and qualitative outcomes. These results provide strong evidence of the effectiveness of improving veterinarian and producer partnerships by establishing formal Veterinary Client-Patient Relationships.

The quantitative data from pre- and post-test scores highlight a statistically significant improvement in participants’ knowledge and skills, indicating that the improvement was not due to chance. Further, the large effect size underscores the substantial impact of the workshops on participants’ learning outcomes. The marked increase in test scores demonstrates participants’ positive reception of the educational content. Qualitative data reflects the likelihood of producers effectively integrating that knowledge into practice. Notably, there were substantial increases in confidence in applying communication strategies, including listing the advantages and consequences of establishing a VCPR, understanding its impact on access to pharmaceuticals and medicated feed, and identifying limitations to veterinary care in the absence of a VCPR. The results also showed significant improvements in understanding the newly revised Feed Directive and recognizing the acceptable use of telemedicine. These knowledge gains and attitude shifts could lead to more informed decision-making and better compliance with veterinary regulations, ultimately improving animal health outcomes.

It is important to note that producers scored lower on a post-training assessment covering the full implementation of the U.S. Food and Drug Administration’s Veterinary Feed Directive than they did on the pre-training assessment. One possible explanation for this decline is that the training made participants aware of the updated regulations. As a result, their initial confidence, based on familiarity with an earlier implementation of the Directive, may have been replaced by uncertainty about the updated regulations. This shift in awareness could have led to lower post-training scores, as learners began to question or reassess their prior knowledge in light of the new information.

Besides improving learning outcomes and confidence levels, the workshops also compelled seven producers to create formal Veterinary-Client Patient Relationships, demonstrating a direct and practical application of acquired knowledge. Additionally, six livestock producers expressed their intention to establish a Veterinary Client-Patient Relationship (VCPR) in the near future, indicating a positive shift in attitudes toward improving herd health through veterinary partnerships.

The lessons developed through this project provide critical educational resources that fill a gap in existing online materials designed for agriculture professionals. The availability of these educational resources through the Extension Foundation and the project website broadens the dissemination of the lessons, both statewide and nationwide, to enhance the project’s long-term sustainability. To date, approximately 1,200 active users, representing seven countries, have engaged with the workshop curriculum on the project website. Three individuals have completed the workshop courses via the Extension website, and another is in process. Collaboration with national organizations and universities eliminates duplication of efforts, leverages funding received to benefit all stakeholders involved, and elevates competency among producers and veterinarians serving the agricultural industry across the United States.

The study results underscore the potential of targeted educational outreach to strengthen veterinarian-producer relationships. However, the study has limitations. The study has a small sample size, which affects both the statistical power and generalizability of the findings. Although the results demonstrated statistically significant improvements in participants’ knowledge and confidence, the limited number of livestock producers (*n* = 26) who completed both pre- and post-tests restricts the ability to draw broad conclusions. Several constraints contributed to the small sample size in this study. One of the primary barriers was the time commitment required to attend a full-day workshop, which proved challenging for many producers who held off-farm employment. Although recruitment efforts targeted underserved producers through established networks, such as Prairie View A&M University and 100 Ranchers, actual participation was limited. Of the 45 individuals who attended the workshops, only 26 were livestock producers—the study’s target population. Veterinarian participation was also low, with only four attending the workshops and none completing pre- or post-tests, further reducing the dataset available for analysis. These logistical and demographic challenges collectively constrained the sample size and impacted the study’s overall reach.

Furthermore, only 27% of producers completed the process, suggesting that barriers beyond financial, such as limited access to veterinarians, lack of motivation, or administrative challenges, may have played a more significant role in limiting participation. While the $250 incentive may have influenced some of the seven producers who established a VCPR, the absence of comparative data makes it difficult to assess its true impact. A more rigorous evaluation design, such as a randomized controlled trial or pre-post comparison, would be necessary to determine the effectiveness of financial incentives. Further research is also needed to identify the underlying factors that hinder VCPR establishment. Additionally, future studies should explore the long-term effects of VCPRs on herd health, as well as their influence on the sustainability and profitability of small and medium-sized livestock operations. Such evidence could inform strategies to support producers in creating and maintaining effective veterinary partnerships.

While targeted educational outreach demonstrates strong potential for strengthening veterinarian-producer relationships, addressing current limitations, such as time constraints and follow-through, and investigating the long-term effects of established Veterinary Client-Patient Relationships (VCPRs) will be essential. Future efforts should focus on sustaining these partnerships through continued education, support mechanisms, and collaborative engagement. Doing so will help ensure that producer-veterinarian relationships contribute meaningfully to the long-term sustainability, animal health outcomes, and economic viability of small to mid-sized livestock operations.

## Data Availability

The datasets presented in this study can be found in online repositories. The names of the repository/repositories and accession number(s) can be found in the article/Supplementary material. The instruments used to conduct this study were developed by the Center for Educational Technologies at Texas A&M University RRID:SCR_022691. Ritter, N.L. (2022). Pre and Post Assessment for Creating Partnerships Curriculum. https://hdl.handle.net/1969.1/1584125. Ritter, N.L. (2022). De-Identified-Data-Pre-and-Post-Workshop-Creating-Partnerships-Training. https://hdl.handle.net/1969.1/1584124.
