# Supplementary material for: Creating partnerships between veterinarians and underserved producers: getting started with establishing veterinary client–patient relationships
Source: Front Vet Sci. 2025 Jul 9;12:1595301. doi: 10.3389/fvets.2025.1595301 (PMC12285527; doi:10.3389/fvets.2025.1595301)

**Appendix A**

**Recruitment Flyer**

This appendix provides the producer and veterinarian recruitment flyers created by Prairie View A&M University. These flyers were distributed via the PVAMU website, county extension agents, and veterinarian professional organizations.


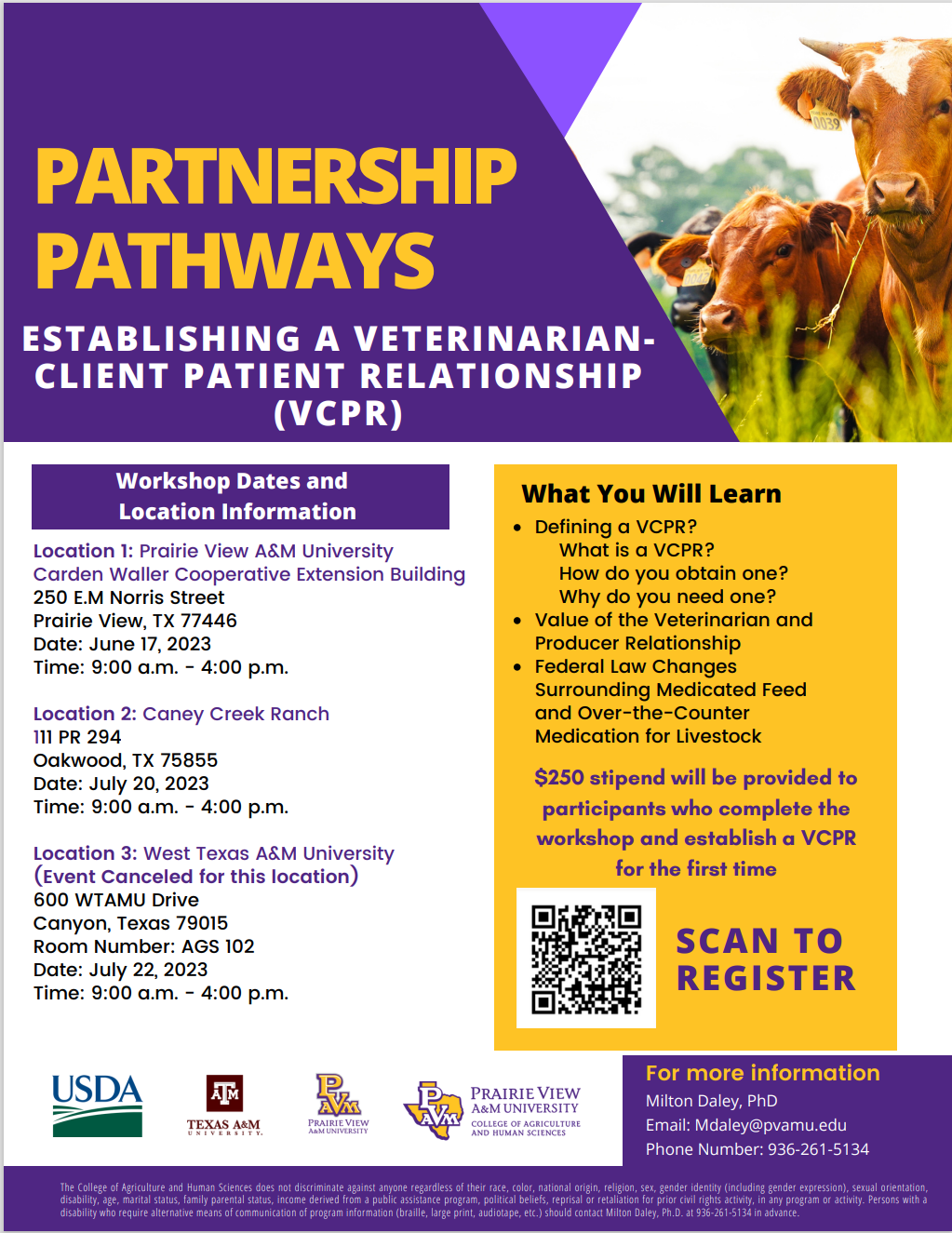


**Appendix B**

**Veterinarian Client Patient Relationship Validation Form**

This appendix provides the Veterinarian Client Patient Relationship validation form. Producers were provided these forms at the workshops and asked to complete and return them within two months.


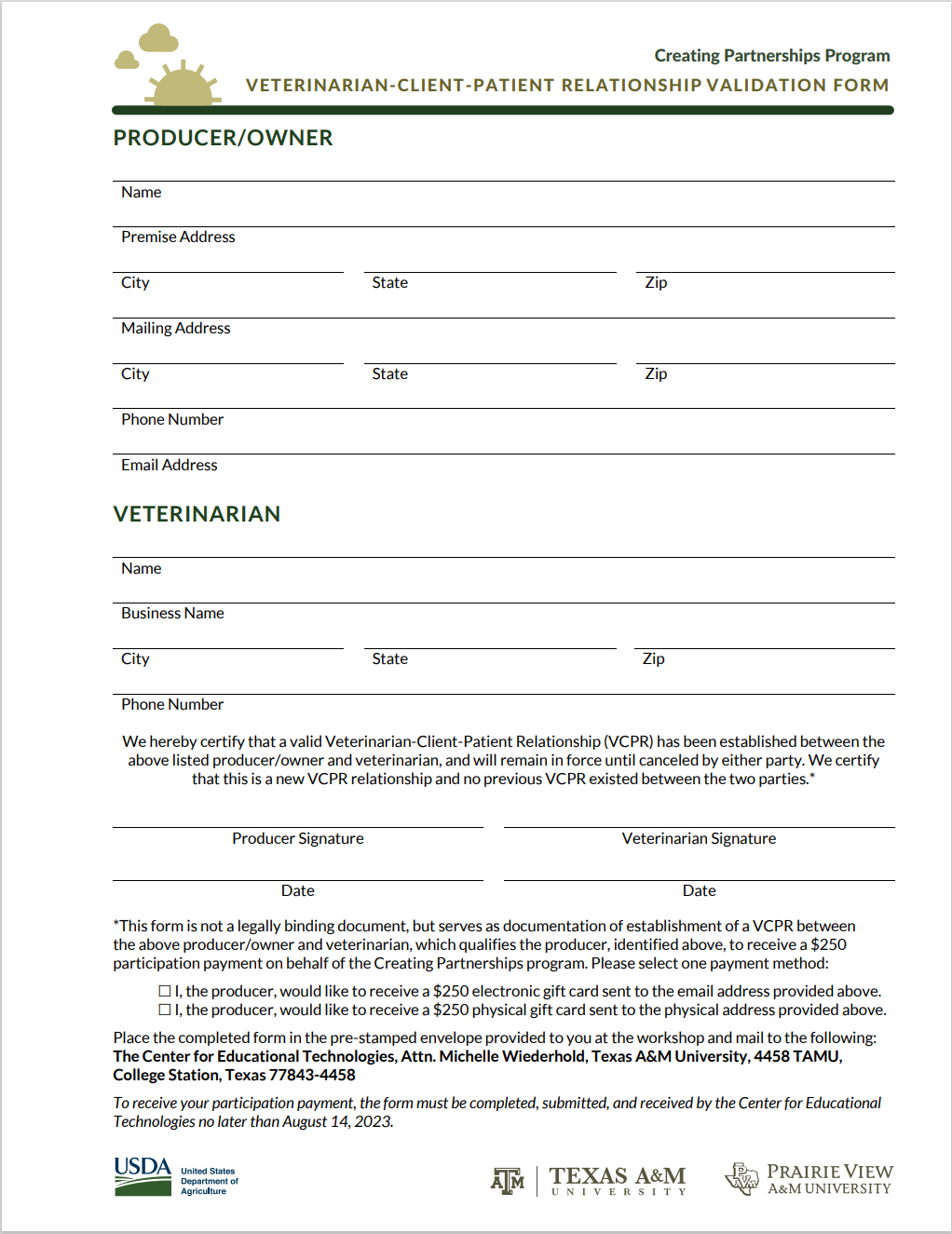


**Appendix C**

**Veterinarian and Producer Workshop Survey**

This appendix provides the workshop survey designed to measure the effectiveness in increasing knowledge and confidence related to veterinary and producer partnerships. The pre and post-surveys for producers and veterinarians include identical questions.


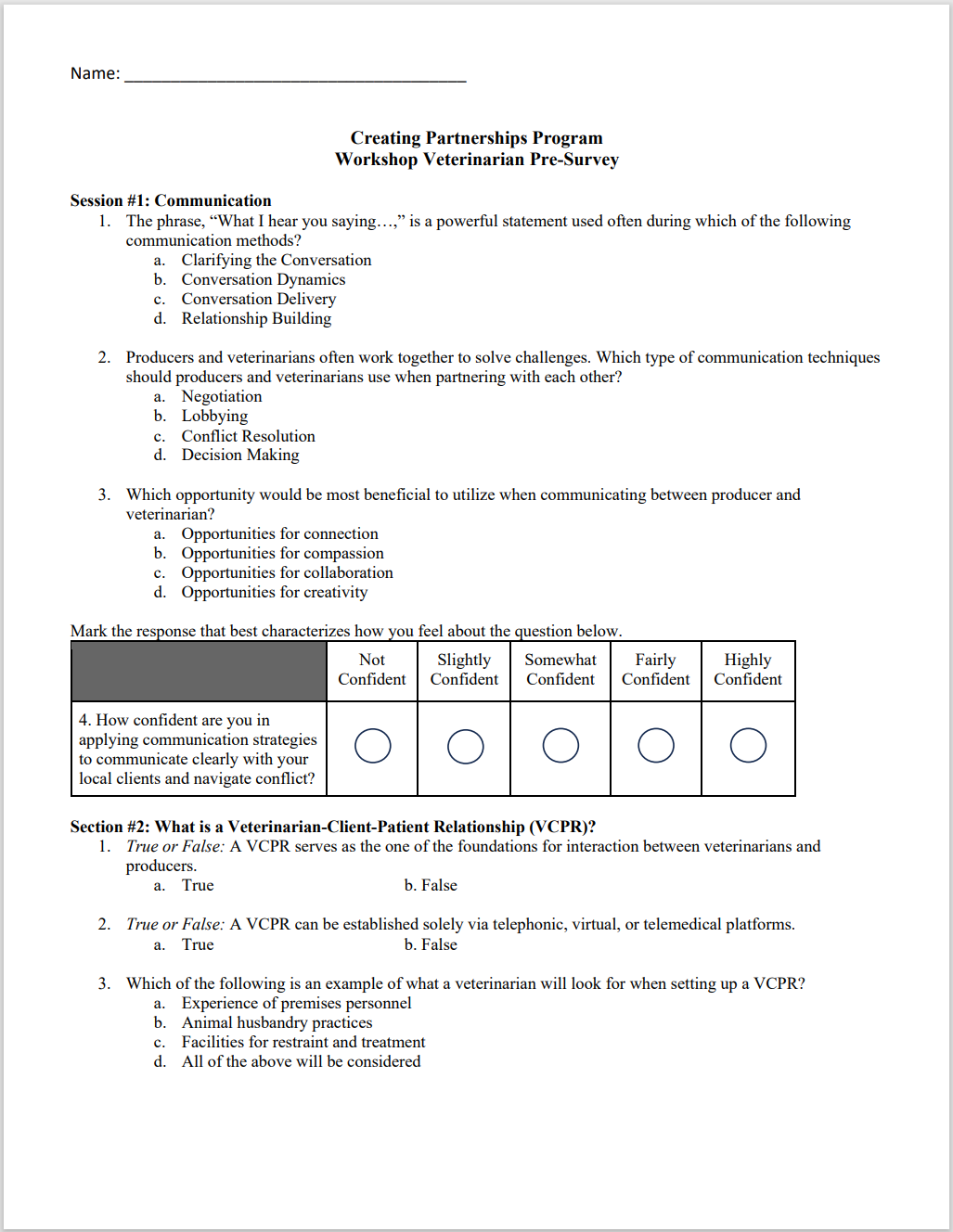


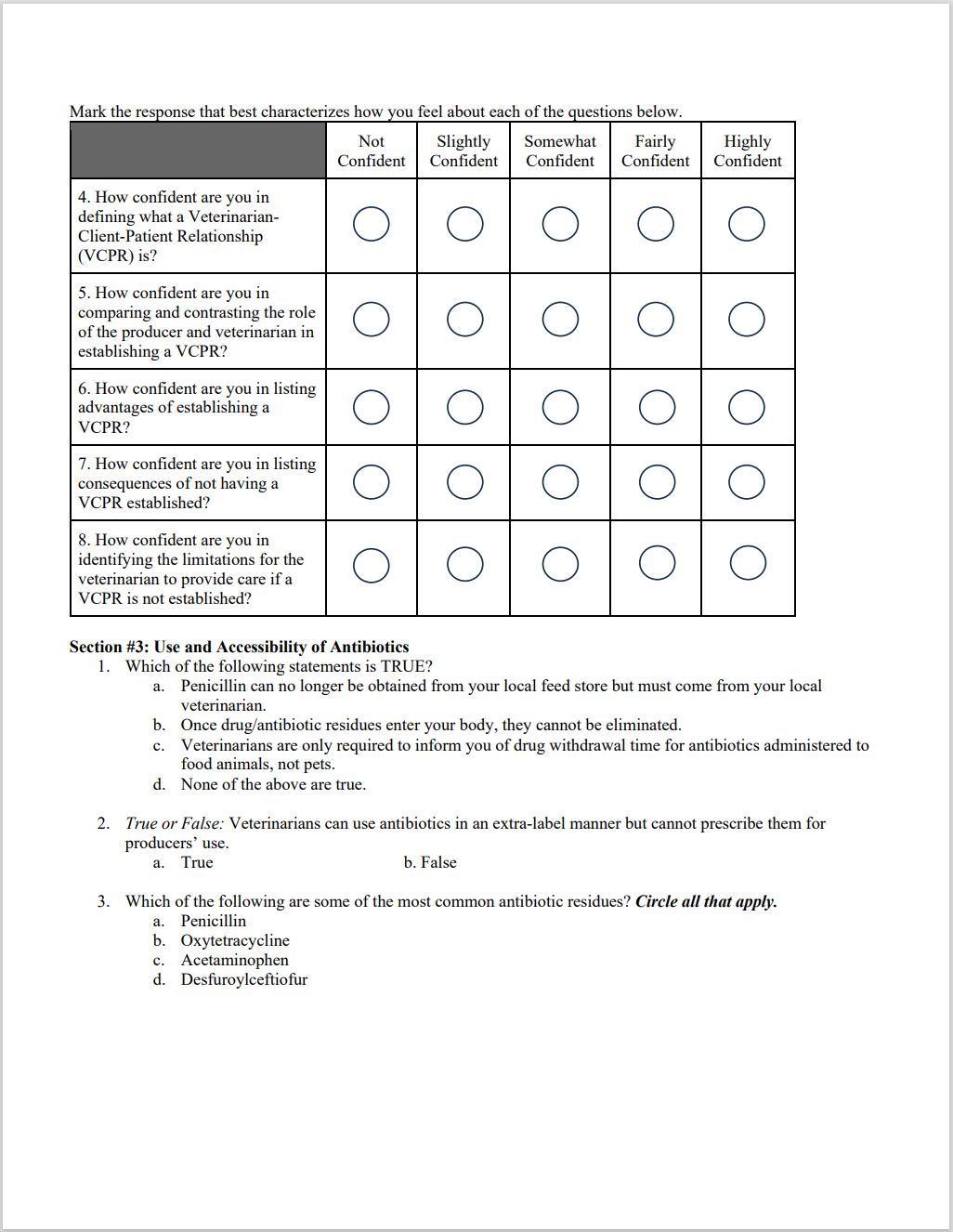


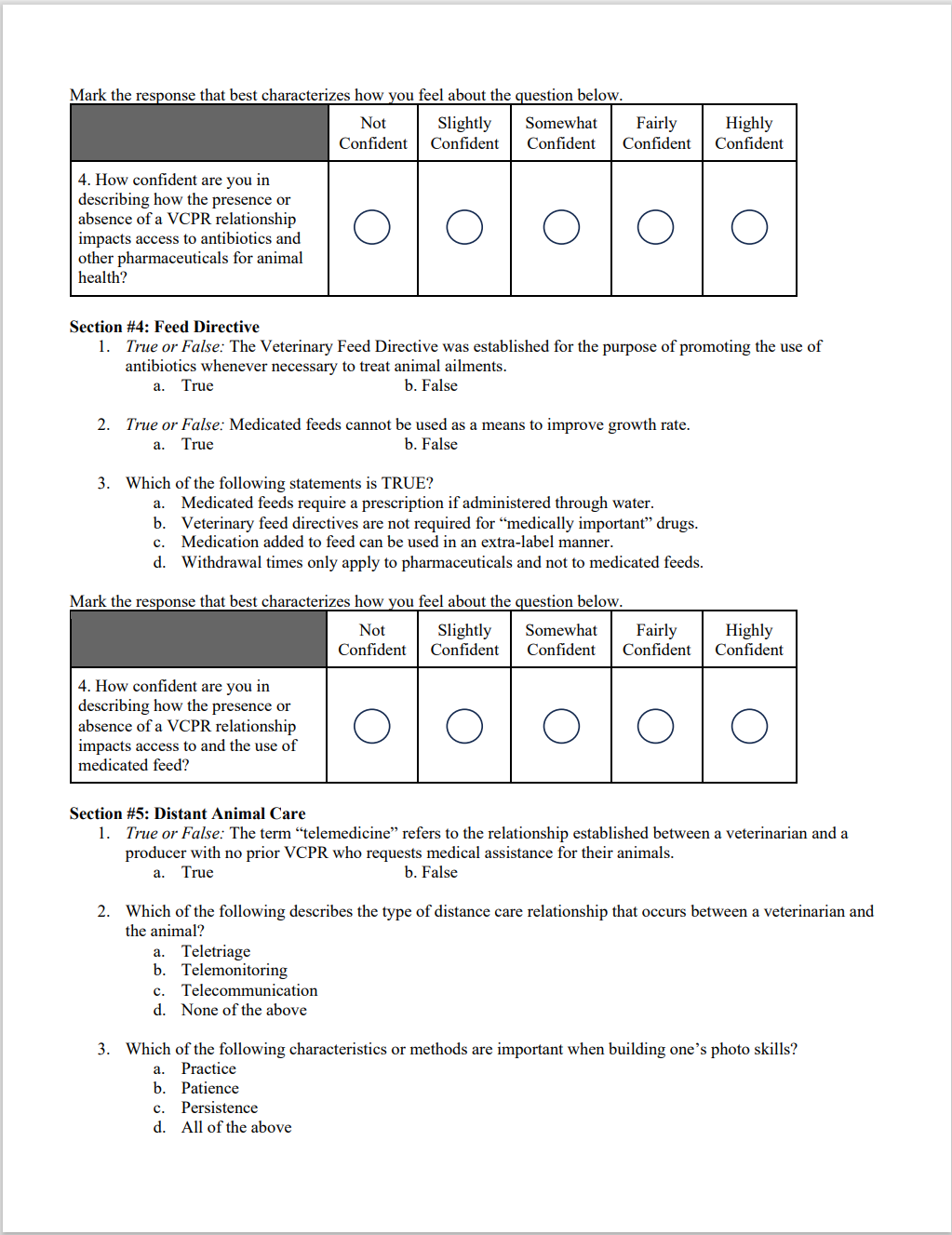


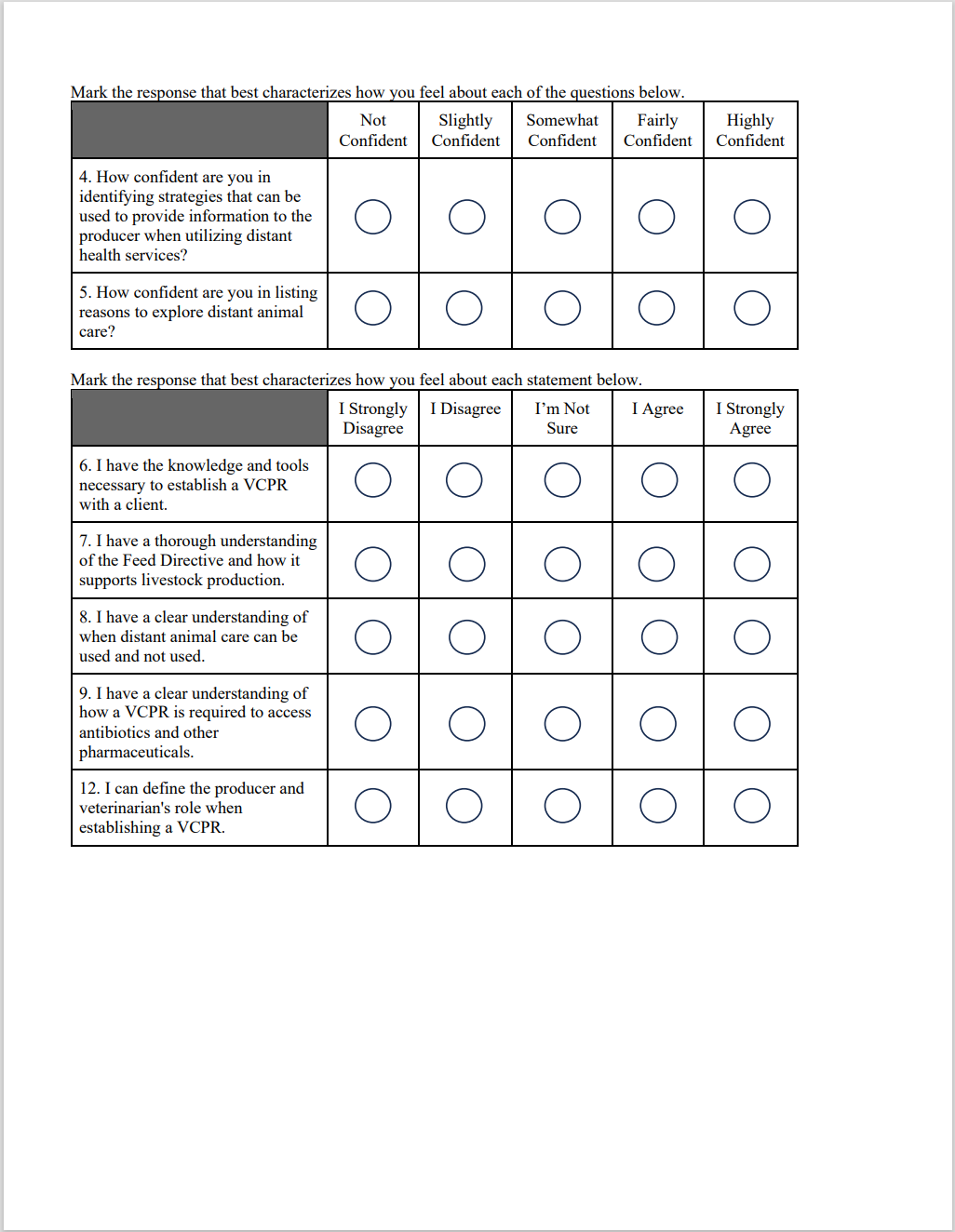

Supplement: Supplementary file 1 [file Data_Sheet_1.docx]
